# The role and significance of potential lipid-binding regions in the mitochondrial protein import motor: an in-depth in silico study

**DOI:** 10.1007/s13205-015-0310-9

**Published:** 2015-05-23

**Authors:** Rob C. A. Keller

**Affiliations:** Section Chemistry, Charlemagne College, Wilhelminastraat 13-15, 6524 AJ Nijmegen, The Netherlands

**Keywords:** Lipid-binding regions, Protein–lipid interactions, Hydrophobic moment plot, Motor protein, Protein translocation, Bioinformatics

## Abstract

Over the last two decades, an impressive progress has been made in the identification of novel factors in the translocation machineries of the mitochondrial protein import and their possible roles. The role of lipids and possible protein–lipids interactions remains a relatively unexplored territory. Investigating the role of potential lipid-binding regions in the sub-units of the mitochondrial motor might help to shed some more light in our understanding of protein–lipid interactions mechanistically. Bioinformatics results seem to indicate multiple potential lipid-binding regions in each of the sub-units. The subsequent characterization of some of those regions in silico provides insight into the mechanistic functioning of this intriguing and essential part of the protein translocation machinery. Details about the way the regions interact with phospholipids were found by the use of Monte Carlo simulations. For example, Pam18 contains one possible transmembrane region and two tilted surface bound conformations upon interaction with phospholipids. The results demonstrate that the presented bioinformatics approach might be useful in an attempt to expand the knowledge of the possible role of protein–lipid interactions in the mitochondrial protein translocation process.

**Electronic supplementary material:**

The online version of this article (doi:10.1007/s13205-015-0310-9) contains supplementary material, which is available to authorized users.

## Introduction

Over the last three decades, multiple types of translocation machineries have been identified in cells (Bohnsack and Schleiff 2010). A motor protein or motor protein machine seems to play a central role in each of those translocation systems. For example in the Sec machinery, the system that facilitates the translocation across and into the prokaryotic membrane is SecA (Vrontou and Economou 2004). While across an endoplasmic reticulum membrane and a mitochondrial membrane, the identified motor proteins are BiP and mtHsp70, respectively (Tomkiewicz et al. 2007). There are, however, important differences in the way of operation. In the Sec system, the motor function is performed by one protein, i.e., SecA (Papanikolau et al. 2007) while in the mitochondrial protein import machinery mtHsp70 is one of the subunits of the matrix import motor (Kang et al. 1990). The identification of other sub-units like Pam18 (D’Silva et al. 2003; Mokranjac et al. 2003; Truscott et al. 2003) substantially increased the understanding of the presequence translocase-associated motor (PAM) of the mitochondrial inner membrane. Recently, new interesting details have been identified regarding the dynamic exchange of Pam18, one of the subunits of the motor-dependent mitochondrial protein translocation, at the motor (Schulz and Rehling 2014).

SecA is one of the best studied proteins in the field of protein translocation. It has not only been identified as a protein that is an essential part of the Sec system (Cabelli et al. 1988) with a well-regulated ATPase activity (Lill et al. 1990) but was also recognized as the protein translocation motor protein in the Sec protein translocation machinery as well (see Vrontou and Economou 2004 for a review). It has been demonstrated that SecA can not only bind but also insert into anionic phospholipid containing membranes (Ulbrandt et al. 1992; Keller et al. 1995). In terms of a mechanistic understanding of these observations, it is interesting to note that a number of possible lipid-binding regions in SecA have been identified (Keller 2011a).

In the same report, multiple lipid-binding regions were found for mtHsp70 (Keller 2011a), a protein with ATPase activity involved in the mitochondrial protein import (Schneider et al. 1996). So at least one component of the protein import motor in the mitochondrial protein translocation machinery exhibits potential lipid-binding regions, just like in SecA (Keller 2011a). Tim44 is a peripheral membrane protein that is able to bind to anionic phospholipids (Weiss et al. 1999). It has been demonstrated that a region in the N-terminal part of the C-terminal domain of this protein is involved in the membrane binding of Tim44 (Marom et al. 2009). Pam18 and Pam16 also known as Tim14 and Tim16 have been studied in great detail (see for example Mokranjac et al. 2006) and it has been suggested that Pam18 contains a transmembrane helix while the closely related Pam16 contains a hydrophobic N-terminus instead. Additionally, a recent report with interesting new details regarding the motor-dependent mitochondrial protein translocation (Schulz and Rehling 2014) triggered the question whether in Pam18 and other subunits of the mitochondrial import motor (novel) helical potential lipid-binding regions can be identified similarly as for mtHsp70. Furthermore, for some of the subunits of the protein import motor, an attempt will be made to give an in-depth in silico characterization of a number of the lipid-binding regions identified. The results will be discussed in terms of the mechanistic implications of our understanding of the functioning of the import motor and the possible role of protein–lipid interactions in this functioning.

## Materials and methods

### Primary and secondary structure

The primary structure of the investigated proteins was obtained from the Uniprot database or from information in the indicated references. Routinely, the extent of helicity was checked either by possible available data and/or by secondary structure prediction using the program SOPMA (Combet et al. 2000) available at http://npsa-pbil.ibcp.fr/. In this study, additional approaches are used for the sake of comparison; see “Secondary structure prediction, crucial start in the search for lipid-binding regions” in “Results” for additional details.

### Determination of lipid-binding potential and Eisenberg plot approach

The main source for the determination of potential lipid-binding regions is the Heliquest software (Gautier et al. 2008); server and additional information are available at the website http://heliquest.ipmc.cnrs.fr/. This was used to obtain data about the mean hydrophobicity ($\langle H\rangle$), the hydrophobic moment (*μH*) and the net charge (*z*). For the analysis, 18-residue windows (selecting α-helix) were used unless stated otherwise. The discrimination factor (*D*) depends on the hydrophobic moment (*μH*) and the net charge (*z*) and is defined according to: *D* = 0.944 ($\langle \mu H\rangle$) + 0.33 (*z*). When the calculation results in a discrimination factor above 0.68, then this region can be considered to be a (potential) lipid-binding helix (Gautier et al. 2008). For the Eisenberg plot approach, both the mean hydrophobicity ($\langle H\rangle$) and the hydrophobic moment (*μH*) were plotted using Heliquest-generated data (see Keller 2011b for further details). Using this approach, the possible surface seeking and transmembrane (TM) helix properties were determined. For the identification of transmembrane regions, a $\langle H\rangle$ value above 0.75 is used (see Keller 2011b for further details).

### Monte Carlo simulations using MCPep

The MCPep server, available at http://bental.tau.ac.il/MCPep/ (Gofman et al. 2012), was used essentially as described before (Keller 2014). The MCPep server used a program implementing a model that allows the performance of Monte Carlo (MC) simulations of the interaction of helical peptides with lipid bilayers and provides a discrimination between the TM and the surface orientation configurations. A typical analysis (see Gofman et al. 2012 for more details) included the input of the corresponding sequence in FASTA format, a membrane width of 30 Å and an RMSD cutoff of 3 Å. See for details about the used anionic lipid content the “Results” section. The number of independent MC runs (three) and the number of MC cycles in each independent run (500,000) were fixed for each analysis.

### Structural modeling

The 3-D structures and corresponding PDB files of a number of proteins studied were generated using I-Tasser (Zhang 2008), available at website http://zhanglab.ccmb.med.umich.edu/I-TASSER/. The ProBLM webserver is used (Kimmett et al. 2014), available at http://compbio.clemson.edu/sapp/problm_webserver/. This program makes use of a geometry-based approach that positioned a membrane protein into a pregenerated lipid membrane and allows manual refinement. A lipid bilayer membrane is used that contains phosphatidylethanolamine (POPE) lipids. Chimera (Pettersen et al. 2004), available at website http://www.cgl.ucsf.edu/chimera/, is used to view the created PDB files in this study.

## Results

### Pam18, an important subunit of the mitochondrial protein motor

Pam18/Tim14 has been identified as an important subunit of the mitochondrial protein motor (see for example Mokranjac et al. 2003). A recent report revealed interesting details of the Pam18 protein in the functioning of the Hsp70 import motor (Schulz and Rehling 2014). Thanks to new developments in bioinformatics approaches, a direct identification of potential helical lipid-binding regions is possible; an important program in this approach is the Heliquest program (Gautier et al. 2008). This program provides data on physical parameters like the mean hydrophobicity ($\langle H\rangle$), the hydrophobic moment (*μH*) and the net charge (*z*). With the use of the Heliquest program (Gautier et al. 2008) and the Heliquest-generated Eisenberg plot (Keller 2011b), a number of potential lipid-binding regions can be identified in Pam18/Tim14 (see Table 1). Two novel lipid-binding regions AA 120–137 and AA 151–168 were identified with the so-called lipid-binding discrimination factor *D* (see Gautier et al. 2008 and the “Materials and methods” section for more details on this); in both cases, the value was above 0.68 (1.59 and 0.78, respectively). An additional lipid-binding region AA 66–83 was identified using the mean hydrophobicity according to the Heliquest-generated Eisenberg plot approach (Keller 2011b) and corresponded to a transmembrane region (Table 1 for further details); the mean hydrophobicity $\langle H\rangle$ was above 0.75 as the threshold value for a typical transmembrane helix. This latter finding corresponds well with the current notion of the presence of a transmembrane helix in Pam18 (see Mokranjac et al. 2006). The results for Pam18 correlate intriguingly well with the finding that also in the Sec system for the motor protein SecA lipid-binding regions were identified with the use of the Heliquest program (Keller 2011a). Furthermore, it is interesting to note that for SecG, a membrane protein involved in the SecYEG complex of the Sec system, a lipid-binding region, was found with the Heliquest program as well as a transmembrane helix as identified with the Heliquest-generated Eisenberg approach (Keller 2013) in a way that resembles that of Pam18. It is tempting to speculate that the reported SecG inversion during protein translocation (Morita et al. 2012) might give a mechanistic explanation for the involvement of Pam18 in the motor functioning of the mitochondrial protein import motor. The results as depicted in Table 1 are predictions, as discussed previously (see Keller 2011b). The confidence for correctly predicting lipid-binding regions is more than 90 %. This accuracy is based on comparison of predictions with well-known and well-described experimental data (see also Gautier et al. 2008 and Keller 2014). It has to be noted that most of the time only novel lipid-binding regions can be expected by the use of the lipid-binding discrimination factor as determined by Heliquest; the possible presence of TM helices as determined by the use of the Heliquest-based Eisenberg plot approach is one of the many ways to identify TM helices. It has been shown, however, that this approach is quite accurate as well (see Keller 2013).

**Table 1:** The lipid-binding region (LBR) search of the subunits of the mitochondrial protein translocation motor

Name	Sequence	*z*	$\langle H\rangle$	$\langle \mu H\rangle$	LBR
Pam18 (66–83)	VITGFGAFLTLYFTAGAY	0	0.857	0.242	TM
Pam18 (120–137)	TENTLTKKKLKEVHRKIM	4	0.040	0.284	*D*
Pam18 (151–168)	ATKINEAKDFLEKRGISK	2	0.017	0.131	*D*
Pam16 (5–22)	AFIQVIITGTQVFGKAFA	1	0.735	0.405	*D*/TM^a^
Pam16 (92–109)	GGSFYLQSKVYRAAERLK	3	0.223	0.256	*D*
Pam16 (107–124)	RLKWELAQREKNAKAKAG	4	0.066	0.091	*D*
Pam17 (4–21)	PSVTAAALRSTATTLPLR	2	0.441	0.094	*D*
Pam17 (52–69)	VGSSLFTALLGCNVSWAY	0	0.791	0.235	TM
Pam17 (87–104)	LTVISAGIIASGALGYLL	0	0.861	0.080	TM
Pam17 (112–129)	VFKLSHNQQLAQFNNKNK	3	0.143	0.162	*D*
Pam17 (163–180)	KEYKQWLRDCHAYAKKAK	4	0.049	0.525	*D*
Tim44 (83–100)	GESEAYKKAREAYLKAQR	2	−0.128	0.273	*D*
Tim44 (94–110)	AYLKAQRGSTIVGKTLKK	5	0.182	0.252	*D*
Tim44 (126–143)	SELGKNTRKAAAATAKKL	4	−0.043	0.386	*D*
Tim44 (180–197)	RRLKRERDLASGKRHRAV	6	−0.229	0.184	*D*
Tim44 (217–235)	SFGKKVEDFKEKTVVGRS	2	0.022	0.245	*D*
Tim44 (226–243)	KEKTVVGRSIQSLKNKLW	4	0.202	0.328	*D*
Tim44 (301–318)	ILEAYVKGDVKVLKKWFS	2	0.487	0.550	*D/S* ^a^

The prediction (and in this case a positive identification of an LBR) is based on either the Heliquest lipid-binding discrimination factor (*D*) or the Heliquest data-generated Eisenberg plot approach to determine the presence of a possible surface seeking helix (*S*) or transmembrane helix (TM)

^a^According to the Heliquest data-generated Eisenberg plot approach, these indicated regions are in close vicinity of the areas of a surface seeking or transmembrane protein

### Pam16, a closely related subunit of the mitochondrial import motor

The protein import motor in mitochondrial protein translocation is a proteinaceous complex with multiple subunits. Pam16 is another subunit of the heat-shock protein 70 (Hsp70)-based import motor (Frazier et al. 2004). Currently, it is believed that Pam18 and Pam16 form a heterodimer and their interaction plays a critical role in the functioning of the import motor (Pais et al. 2011). Just as in Pam18, two novel lipid-binding regions AA 92–109 and AA 107–124 were identified (see Table 1 for details). The lipid-binding discrimination factor *D* was found in both cases to be above 0.68 (1.23 and 1.41, respectively). An additional lipid-binding region AA 5–22 was identified and, according to the mean hydrophobicity found ($\langle H\rangle$ = 0.735) which is close to the threshold value of 0.75, corresponded possibly to a transmembrane helix region. This corresponds well with the earlier described findings that Pam16 contains no TM helix but a hydrophobic region instead (Mokranjac et al. 2006). This issue is analyzed further using other bioinformatics tools and discussed later on, see “Some additional characterizations of lipid-binding regions of other subunits” in “Results” and supplementary materials Fig. S2 for helical wheel plots.

### Pam17, another membrane-associated subunit of the import motor

Pam17 is one of the membrane-associated co-chaperones. It interacts with the channel protein Tim23 and thus forms an interaction site between Tim23 and the mitochondrial protein import motor (Hutu et al. 2008). As in Pam16 and Pam18, a number of novel potential helical lipid-binding regions AA 4–21, AA 112–129 and AA 163–180 were identified with the lipid discrimination factor *D*; in all those cases, the value was above 0.68 (0.75, 1.14 and 1.82, respectively) (see Table 1 for details). Additionally, two lipid-binding regions AA 52–69 and AA 87–104 were identified and according to their mean hydrophobicity ($\langle H\rangle$ = 0.791 and 0.861, respectively), they clearly correspond to a transmembrane region according to the threshold value of 0.75 as used in the Heliquest-generated Eisenberg plot approach (Keller 2011b). This corresponds well with earlier findings (Maarse et al. 1994). These two regions will be analyzed and discussed further later on this section. See supplementary materials Fig. S2 for helical wheel plots.

### Tim 44, the adaptor protein of the mitochondrial protein import motor

It was found that an efficient interaction of Tim44 with mtHsp70 is necessary for a proper functioning of the mitochondrial protein import motor (Merlin et al. 1999). Numerous lipid-binding regions were found for Tim44 (see Table 1 for details). Only one region AA 301–318 might, according to the Heliquest-generated Eisenberg plot analysis, be classified as a ‘classical’ surface seeking helix. The mean hydrophobicity $\langle H\rangle$ and the hydrophobic moment $\langle \mu H\rangle$ (0.487 and 0.550, respectively) put this region very close to the surface seeking area in the Heliquest-generated Eisenberg plot, which is defined as positions above the following line $\langle \mu H\rangle$ = 0.645 − 0.324 $\langle H\rangle$ (see Keller 2011b for further details). This region will be analyzed and discussed further later on in this section. See supplementary materials Fig. S2 for helical wheel plots. All other regions fall into the globular protein region of the hydrophobic moment plot and might indicate that Tim44 according to these characteristics be a member of the amphitropic protein family (Keller 2014). Indeed, this corresponds nicely with the earlier suggestion that Tim44 is a peripheral membrane protein (Weiss et al. 1999). Region AA 226–243 corresponds nicely with one of the earlier identified lipid regions, the so-called A1 helix of Tim44 (Marom et al. 2009). The A2 helix as described in the same paper seems to be missed by the Heliquest-based lipid-binding region search approach. However, as discussed previously (Keller 2014) occasionally matters like charge neutralization might be involved. Indeed if one of the, for example, glutamic acids in this region is neutralized upon binding to negatively charged phospholipids, this region can act as a potential helical lipid-binding region. All other regions as identified here are novel, as indicated before (Marom et al. 2009); the existence of other regions besides the A1 and A2 helix involved in lipid binding could not be excluded and might be identified in the present study.

### Secondary structure prediction, crucial start in the search for lipid-binding regions

The identification of lipid-binding regions is focused on the helical parts of proteins like the Pam18. This is important because the prerequisite of the (manual) use of Heliquest is that the region must be helical. The first step in the search for possible lipid-binding regions involves a secondary structure analysis using prediction programs such as SOPMA (Combet et al. 2000) as used in previous studies routinely (see for example Keller 2014). In Table 2, a comparison of the secondary structure analysis is made between different approaches. It is clear that overall a method like PSIPRED (McGuffin et al. 2000) available at http://bioinf.cs.ucl.ac.uk/psipred/ gave comparable results to SOPMA. The same is true for another frequently used prediction method JPred3 (Cole et al. 2008) available at http://www.compbio.dundee.ac.uk/www-jpred/. Interestingly, I-TASSER (Zhang 2008) available at http://zhanglab.ccmb.med.umich.edu/I-TASSER/, a bioinformatics method that predicts 3D structures of protein molecules from their primary sequence and generates secondary structure predictions as well, gives very similar results. One of the most recent protein secondary structure prediction methods is CONCORD (Wei et al. 2012) available at http://helios.princeton.edu/CONCORD, a method based on a mixed integer linear optimization of seven secondary structure prediction methods. This method was performed in a comparable way to the single methods. The paper describing the CONCORD method goes into the performance and accuracy in great detail (Wei et al. 2012) and together with the excellent discussion by Rost (2001) it has been indicated that every individual method for protein secondary structure prediction has a theoretical limit of 88 % accuracy. Although the results in Table 2 can only be used for indicative purposes when it comes to a comparison of the performance of the different methods, it is clear that the overall results justify the conclusion that the identified potential lipid-binding regions are most likely helical. As indicated above, SOPMA is used in earlier studies (see for example Keller 2011a). It is a straight forward, fast and easy to use approach with overall reliable results. The Heliquest approach requires sufficient helical content of the region of interest but allows a wide range; in other words, it is not extremely important to know whether the helical content is 60 or 89 %. However, the result of Tim14 region AA 66–83 demonstrates the potential danger that you can miss a region because of a (too) low helical content based on one single prediction method. The results in Table 2 seem to indicate that at least two different methods need to be used to minimize incidental misinterpretation. Based on the results and (earlier) experiences with the methods used, SOPMA and CONCORD are good candidates.

**Table 2:** Comparison of different secondary structure prediction methods with the method used routinely in this study SOPMA

Name	SOPMA	PsiPred	JPred3	I-TASSER	CONCORD
Pam18 (66–83)	17	100	50	89	83
Pam18 (120–137)	83	67	61	67	67
Pam18 (151–168)	72	78	78	61	72
Pam16 (5–22)	56	100	45	100	100
Pam16 (92–109)	61	72	50	83	83
Pam16 (107–124)	94	89	40	94	94
Pam17 (4–21)	56	72	28	22	50
Pam17 (52–69)	56	100	100	100	100
Pam17 (87–104)	61	78	100	100	100
Pam17 (112–129)	83	94	78	100	100
Pam17 (163–180)	78	100	100	100	100
Tim44 (83–100)	89	83	100	100	78
Tim44 (94–110)	61	100	100	100	83
Tim44 (126–143)	83	100	83	100	100
Tim44 (180–197)	67	50	50	55	56
Tim44 (217–235)	61	72	67	94	67
Tim44 (226–243)	78	56	72	94	72
Tim44 (301–318)	83	78	78	83	89

Indicated values are the α-helical content (in %)

### Further characterization of the lipid-binding regions of Pam18

**Fig. 1:**
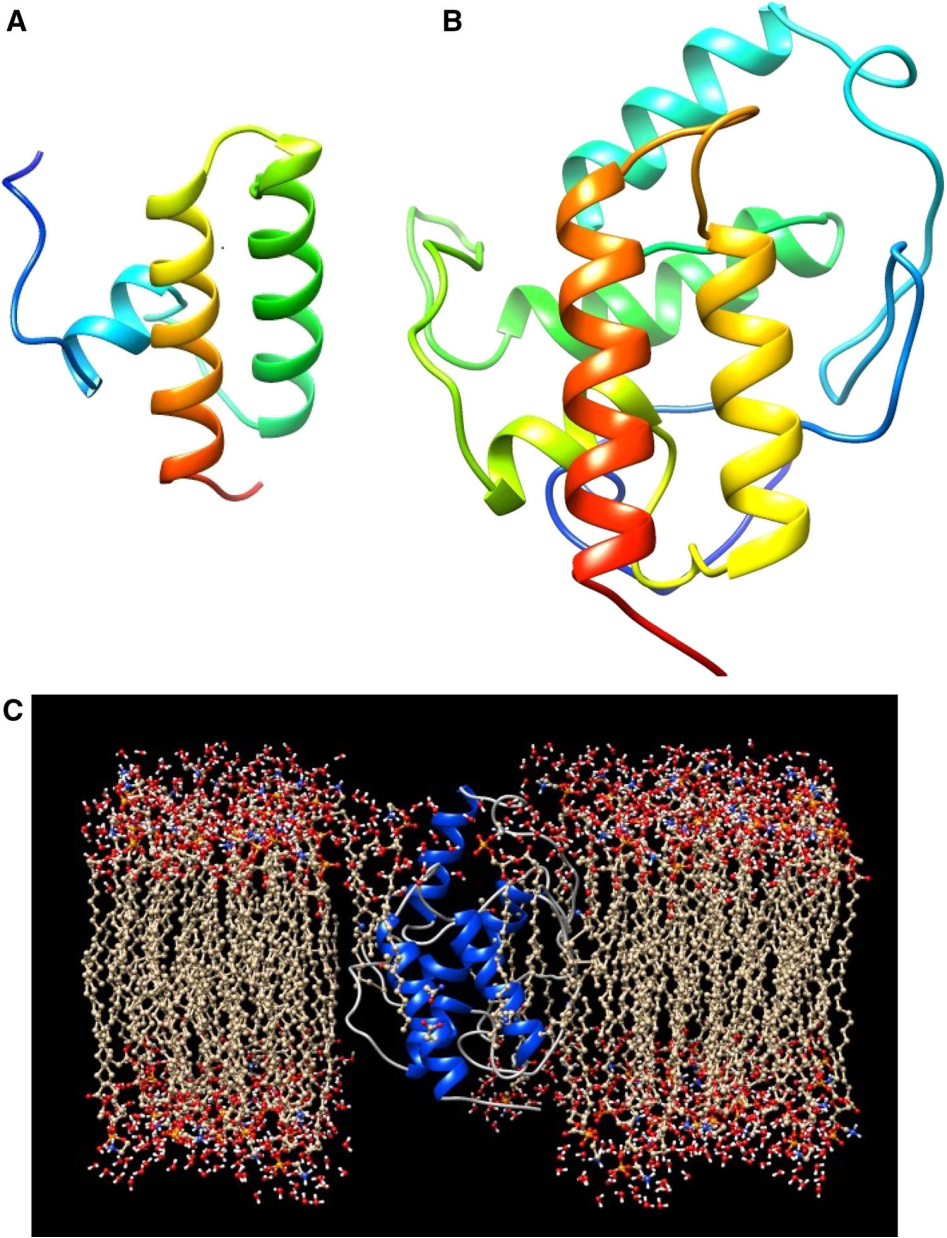
Ribbon presentation of the truncated Pam18, PDB entry 2GUZ (**a**). The creation of the PDB file of the full Pam18 by I-TASSER (**b**). This PDB file is used to view the full protein embedded in a phosphatidylethanolamine (POPE) membrane with the use of the ProBLM server (**c**). In **a** and **b**, the N-termini are depicted in *blue* and towards the C-termini the color turns into *red*

The only crystal structure of Pam18/Tim14 determined using X-ray diffraction (deposited with PDB entry 2GUZ) is unfortunately based on the AA 99–168 fragment of the protein, including the so-called J-domain (region AA 112–168) but lacking the transmembrane helix (Mokranjac et al. 2006); see Fig. 1a. To get an idea how the complete protein looks like, the whole sequence is analyzed using the program I-TASSER (see Fig. 1b). The part that corresponds to the AA 99–168 fragment (Fig. 1b) looks similar to that reported before (Mokranjac et al. 2006), which is no real surprise since the MC approach used by the I-TASSER program makes use of the PDB entry 2GUZ. It is clear that in the absence of lipids or even detergents, it is hard to estimate how meaningful in this model the position of the transmembrane helix really is. To get some idea about this, the new ProBLM webserver is used (Kimmett et al. 2014). This new program makes use of a geometry-based approach that inserts a membrane protein with an available protein coordinate file into a pregenerated lipid membrane. The PDB file is used which was created with the I-TASSER program (the model as depicted in Fig. 1b) and this is used as input for the ProBLM software. In the result as depicted in Fig. 1c, a lipid bilayer membrane is used that contains phosphatidylethanolamine (POPE) lipids. Obviously, the result requires further handling, for example, a minimization step using molecular dynamics tools (see, for more details and an initial result; supplementary materials). According to the makers of this program, single-spanning alpha-helical transmembrane proteins can tend to give an axis that may be tilted with respect to the membrane normal (Kimmett et al. 2014). Although manual adjustment is possible, it is not done here since to which extent it is valid to look closer at the helical lipid-binding regions in this model is somewhat questionable since two of the identified lipid-binding regions AA 120–137 and AA 151–168 are anionic phospholipid dependent (since the Heliquest approach indicates a preference of the identified regions for anionic phospholipid membranes and the protein has been demonstrated to be anionic phospholipid dependent for its binding (Weiss et al. 1999; Maron et al. 2009) and unfortunately anionic lipids cannot be used in the ProBLM software. However, the corresponding PDB files are available for those who want to submit the conformations to a further analysis (see supplement). The model as depicted in Fig. 1c indicates that Pam18 and the helical regions can be positioned in the membrane and is included for indicative purposes.

**Fig. 2:**
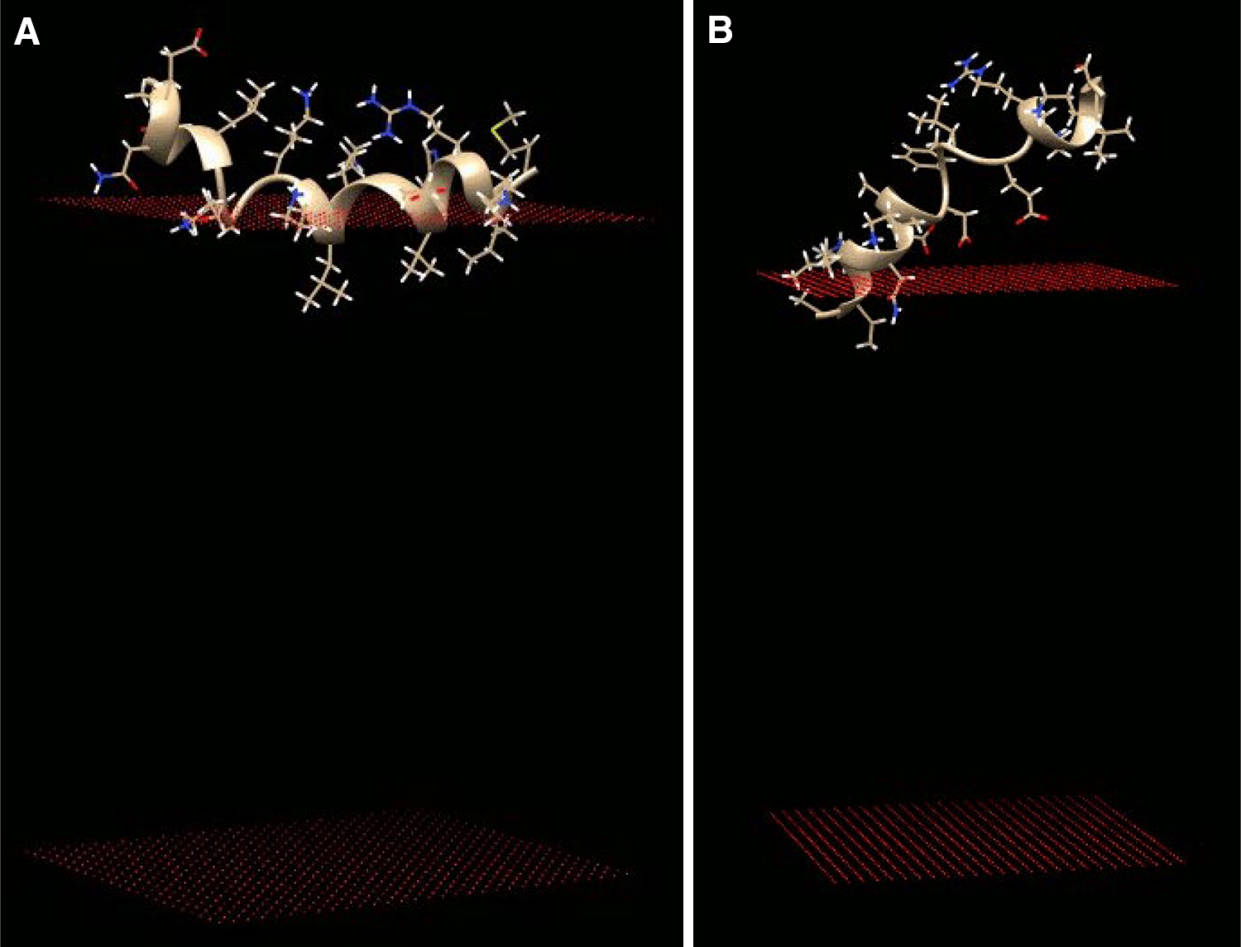
MCPep results of Pam14 lipid-binding regions AA 120–137 (**a**) and AA 151–168 (**b**). Monte Carlo simulations of peptide interactions with membranes containing 20 % anionic phospholipids (phosphatidylglycerol, PG) are depicted (see “Materials and methods” for details)

More details can be found by focusing on the individual lipid-binding regions. The two identified lipid-binding regions AA 120–137 and AA 151–168 were analyzed further by the MCPep program. This MC approach allows a simulation of the two helical peptides upon possible binding to both neutral and negatively charged phospholipids. For neutral membranes, the MCPep program was unable to identify transmembrane or surface bound configurations (data not shown). The results for the two helical lipid-binding regions AA 120–137 and AA 151–168 with membranes containing 20 % anionic phospholipids are depicted in Fig. 2. The MCPep result of the other region, the transmembrane region AA 66–83, is somewhat complicated. It appeared that although the Heliquest program identified this region as a clear transmembrane region, MCPep was unable to come up with a transmembrane configuration (see supplement Fig. S1). However, taken a closer look at the hydrophobic scale (or better the free energy data) of the amino acids on which the MCPep method is based on (Kessel and Ben-Tal 2002) then it becomes apparent that the tyrosine corresponds, according to this scale, to a very hydrophilic amino acid. Since this particular membrane region contains two tyrosine amino acids, this could very well explain why MCPep cannot put such a region in the hydrophobic core of the membrane. Tyrosine must, however, most likely be considered as a medium hydrophobic amino acid with a polar character, so medium in terms of hydrophobic/hydrophilic (see also supplement for further details on this). Indeed when those two amino acids were replaced by a hydrophobic amino acid such as leucine, a perfect transmembrane configuration was found by MCPep (see Fig. S1 in the supplement). The helical wheel plot in Heliquest is identical (see supplement); the mean hydrophobicity is even higher (more than 1, data not shown) again leading to the conclusion that we are dealing here with a TM helix. There is, however, another indication that this particular region is not a straightforward and easy to detect transmembrane helix, since the state-of-the-art topology prediction method TOPCONS (Bernsel et al. 2009) had great difficulties in identifying this region (see the supplement for further details). In this respect, it is interesting to note that earlier findings demonstrated that a truncated Pam18/Tim14 protein that lacks this transmembrane region is still functional in vivo (Mokranjac et al. 2006), already indicating the special nature of this TM helix and membrane protein. In conclusion, it seems that Pam18 contains two novel potential helical lipid-binding regions and one possible transmembrane region.

### Some additional characterizations of lipid-binding regions of other subunits

Some issues in the presented results are interesting and important to check somewhat further. For example, the Pam16 region AA 5–22 is according to the Heliquest-generated Eisenberg plot approach (Keller 2011a) closely situated to the membrane protein area in a hydrophobic moment plot, since the mean hydrophobicity $\langle H\rangle$ found (0.735) is close to the threshold value 0.75 as defined by the Heliquest-generated Eisenberg plot approach (Keller 2011a). Indeed, a TOPCONS analysis indicates the likely presence of a transmembrane region at region 2–22 which is remarkably close to the region found with Heliquest (see supplement for further details). These results explain why in the current literature (see for example Bajaj et al. 2014) Pam16 is often depicted without a TM helix and contains a hydrophobic region instead (Mokranjac et al. 2006).

**Fig. 3:**
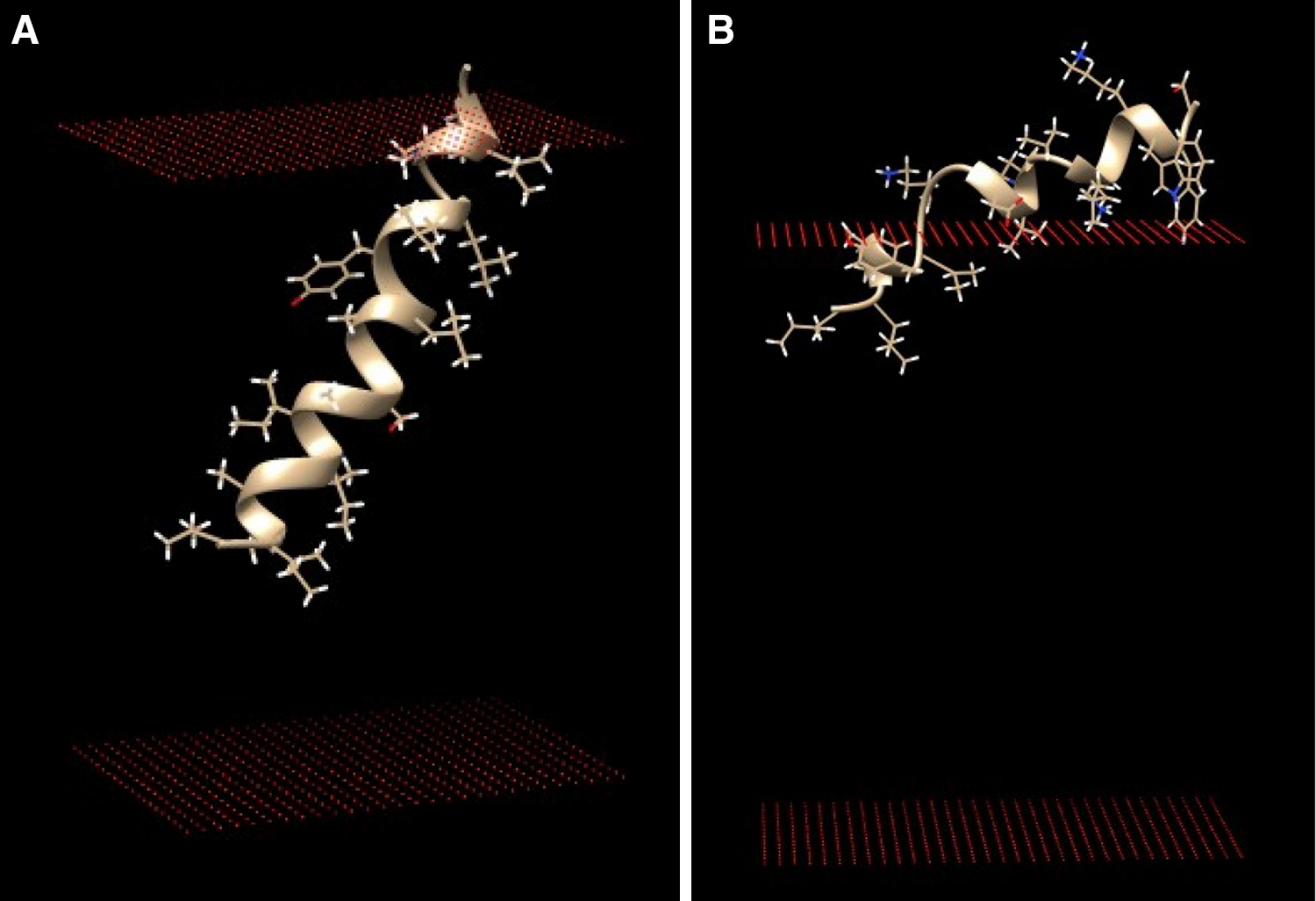
Monte Carlo simulations of peptide interactions with membranes containing 20 % anionic phospholipids (phosphatidylglycerol, PG) corresponding to one of the Pam17 transmembrane regions AA 87–104 (or more precise AA 89–109, see for details “Results” section) (**a**) and the Tim44 surface seeking region AA 301–318 (**b**)

Another interesting example is related to the Pam17 transmembrane regions AA 52–69 and AA 87–104 which were identified as transmembrane helical regions according to the Heliquest approach; both values of $\langle H\rangle$ were found to be above the threshold value of 0.75 (see Table 1). For the AA 87–104, no TM configuration could be found by the use of MCPep (instead a surface configuration was given, data not shown). However, a slightly shifted region AA 89–109 was clearly a transmembrane configuration according to MCPep. This example of the MCPep analysis is depicted in Fig. 3. Indeed the corresponding peptide gave a typical example of a transmembrane helix when inserted in a lipid membrane containing 20 % anionic phospholipids.

Finally in the Tim44, the only example of a typical surface seeking helix was predicted by the Heliquest approach, according to rule that a region needs to be above the following line $\langle \mu H\rangle$ = 0.645 − 0.324 $\langle H\rangle$ (see Table 1). The MCPep result of this surface seeking region AA 301–318 upon interaction with phospholipid membrane containing 20 % anionic phospholipids is depicted in Fig. 3. Indeed the corresponding peptide corresponds to a typical picture of a surface seeking helix upon binding to a membrane containing negatively charged lipids. It is important to note that results found by the MCPep approach confirm the results as found by Heliquest. In other words, the MCPep only will give significant clusters of surface bound or transmembrane configurations when the peptide corresponding to a particular lipid-binding region, according to the calculations of the program, interacts with phospholipids.

## Discussion

It is clear that based on the results as shown in this paper, potential helical lipid-binding regions were found for proteins belonging to the mitochondrial protein translocation motor. For example, it has been demonstrated that Pam18 contains three potential helical lipid-binding regions (Table 1). One of the identified regions corresponds with the well-described presence of a transmembrane helix. The unclear nature and apparent difficulty to predict and characterize this region as described in this paper might correspond to the finding that Tim14 lacking its transmembrane anchor is still functional in vivo (Mokranjac et al. 2006). Two additional novel lipid-binding regions were identified and described here (Table 1). The ProBLM result as depicted in Fig. 1 seems to indicate a possible membrane embedded conformation. Additional works needs to be done to make this result more than useful for indicative purposes; however, further characterization of the three lipid-binding regions using MCPep leads to a more detailed picture of the identified lipid-binding regions, one possible transmembrane region and two tilted surface bound conformations. The MCPep results indicate the possibility to get further in-depth knowledge of the protein–lipid interactions in more mechanistic terms (see for example Figs. 2, 3).

The earlier finding that mtHsp70 contains multiple lipid-binding regions (Keller 2011a) and the presented paper indicates that in the other subunits of the mitochondrial protein import motor possible helical lipid-binding regions can be identified as well. As discussed previously (Keller 2014), the lipid-binding regions are not expected to be conserved (in terms of primary sequence), since they are based on overall physicochemical features (like the mean hydrophobicity $\langle H\rangle$ and charge *z*) of the regions. Although not conserved in terms of primary sequence, lipid-binding regions are found in different organisms for similar proteins, like SecD (Keller 2013) and SecA (Keller 2014). In relation to this, it is perhaps important to stress that methods like Heliquest and MCPep do not allow a closer look at the lipid specificity, although Heliquest can indirectly discriminate between neutral and negatively charged phospholipids by the *z* value in the lipid discrimination factor or as indicated by the researchers behind the Heliquest program (see http://heliquest.ipmc.cnrs.fr/TablePeptide.htm), the lipid-binding prediction correlates well with the “ability of a segment to bind in vitro to large liposomes containing phosphatidylcholine and negatively charged lipids (phosphatidylserine, phosphatidylinositol or phosphatidylglycerol)”. The MCPep allows the user to adapt the amount of anionic phospholipids in the membrane used for the simulations but at least the freely available online version does not allow the introduction of different kind of lipids.

The accuracy of the predictions of potential helical lipid-binding regions is based on comparison of the predictions with numerous data of experimentally demonstrated lipid-binding regions (see Gautier et al. 2008; Keller 2011a for more details). In this respect, it is interesting to note that the finding of such potential helical lipid-binding regions in one of the Hsp70 proteins (Keller 2011a) has recently been substantiated by the experimental evidence for the existence of protein–lipid interactions for human Hsp70 (Mahalka et al. 2014). Intriguingly, similar results were found for the Sec system motor protein SecA (Keller 2011a). There is already some experimental evidence for the existence of a number of those lipid-binding regions in SecA (Breukink et al. 1993, 1995). Recently, an elegant approach demonstrated how it is possible to dissect the role of a lipid-binding region in factors like membrane binding, lipid specificity and channel activities once you focus on one of those lipid-binding regions of SecA (Floyd et al. 2014) and basically confirmed the existence of the predicted N-terminal lipid-binding regions AA 1–21 and AA 14–33 in *E. coli* SecA (Keller 2011a). A substantial number of potential helical lipid-binding regions in a number of subunits of the mitochondrial protein import motor are predicted in this paper and some of those regions were characterized further in silico (see Table 1). Taken this altogether, this could indicate that the identified possible helical lipid-binding regions in the mitochondrial protein import motor might play a novel and active role in the protein translocation of proteins across the mitochondrial membrane. It would be interesting to see if certain lipid-binding regions are involved in a particular stage of the protein translocation process, like in the initial stages as has been suggested for Tim44 (Weiss et al. 1999), while others might be involved in later stages of the process.

The role of anionic phospholipids in the protein translocation across the cytoplasmic membrane using the Sec system is well studied (see for a review van Klompenburg and de Kruijff 1998). In the mitochondrial protein translocation, the number of papers with the phospholipids as focus is limited. Some indirect indications of the involvement of phospholipids are described in the literature. For example, the finding that control of the cardiolipin metabolism and the effect on the assembly of Tim23 and the regulation of the association with PAM are related matters seems to imply an involvement of cardiolipin in the mitochondrial protein sorting (Tamura et al. 2009). The way phosphatidylethanolamine (PE) and cardiolipin (CL) affect the stability of mitochondrial respiratory chain supercomplexes has been studied in quite some detail and the subsequent effect on the inner membrane potential ∆*ψ*, important for protein translocation, was demonstrated (Böttinger et al. 2012). This seems to indicate that one of the roles of (certain) phospholipids is to keep the protein translocation machinery in a protein translocation competent state. There are some indications that specific protein-anionic phospholipid interactions in mitochondrial protein sorting do play a role, as discussed before (Weiss et al. 1999, Marom et al. 2009). For example, it was shown that anionic phospholipids induce a marked conformational change in the mitochondrial presequence (Epand et al. 1986). The interactions of apocytochrome c with anionic phospholipids are studied in great detail (see for example Snel et al. 1994). Interesting to note that all the regions identified to be of importance for apocytochrome c interaction with phospholipids that were determined experimentally could be identified by the Heliquest lipid discrimination factor *D* as well (Keller 2011a). A great step in the elucidation of the role of each component in the mitochondrial protein translocation process is the development of a reconstitution system and preliminary results clearly indicate the necessity for cardiolipin (van der Laan et al. 2007). There is an excellent review published that not only summarized the current status of our knowledge about the lipid involvement in mitochondrial protein translocation but also highlighted some important remaining issues that need to be resolved (Gebert et al. 2011). In this respect, the newly identified possible helical lipid-binding regions in subunits of the mitochondrial protein import motor as presented in this paper might give new tools for further investigations. The observation that in different motor proteins belonging to two different protein translocation systems multiple helical lipid-binding regions can be identified is intriguing enough for a closer look.

## Electronic supplementary material

Supplementary material 1 (DOCX 1710 kb)

## Abbreviations

Hsp: Heat shock protein
Pam: Presequence translocase-associated motor
Tim: Translocase of the inner membrane
PE: phosphatidylethanolamine
