# Supplementary material for: The role and significance of potential lipid-binding regions in the mitochondrial protein import motor: an in-depth in silico study
Source: 3 Biotech. 2015 May 23;5(6):1041–51. doi: 10.1007/s13205-015-0310-9 (PMC4624131; doi:10.1007/s13205-015-0310-9)
Supplement: Supplementary file 1 — Supplementary material 1 (DOCX 1710 kb) [file 13205_2015_310_MOESM1_ESM.docx]

**The role and significance of potential lipid binding regions in the mitochondrial protein import motor: an in-depth *in silico* study.**

Rob C. A. Keller

**Supplementary materials**

This supplement contains additional information and data as indicated in the main text of the paper. Details can be found regarding:

I-Tasser and ProBLM details of Pam18/Tim14

The MCPep results of the AA region of Pam18

The TOPCON results of Pam18/Tim14

Comparison of different hydrophobicity scales

The TOPCON results of Pam16

Helical wheel plots of indicated lipid binding regions as obtained by Heliquest.

I-Tasser and ProBLM details of Pam18/Tim14

As described in the RESULTS section, the additional information belonging to the structural models are given below. For the I-TASSER the following FASTA form is used as input:

>sp|Q07914|TIM14_YEAST Mitochondrial import inner membrane translocase subunit TIM14 OS=Saccharomyces cerevisiae (strain ATCC 204508 / S288c) GN=PAM18 PE=1 SV=1

MSSQSNTGNSIEAPQLPIPGQTNGSANVTVDGAGVNVGIQNGSQGQKTGMDLYFDQALNYMGEHPVITGFGAFLTLYFTAGAYKSISKGLNGGKSTTAFLKGGFDPKMNSKEALQILNLTENTLTKKKLKEVHRKIMLANHPDKGGSPFLATKINEAKDFLEKRGISK

In order to check the models as depicted in the report in detail, the following PDB files can be used. Drag the desired file to your desktop and/or save the desired PDB files on your computer and open them in a program like Chimera (available at <https://www.cgl.ucsf.edu/chimera/>). This way you can look at the structure in full detail.

Pam18 (region 99-168):

Pam18 (region 1-168):

Pam18 (full length) in a POPE bilayer 100/50 dimensions as obtained by proBLM:

The result as obtained by ProBLM (Pam18POPEmembrane.pdb) can be used for further processing. For example a minimization step by a molecular dynamics tool. In this case an energy minimization step is performed by YASARA (http://www.yasara.org/minimizationserver.htm). The pdb file is subjected to the energy minimization step and the result can be viewed by YASARA view (downloadable from http://www.yasara.org/products.htm#view). The result can subsequently be saved as a pdb file and further processed by chimera.


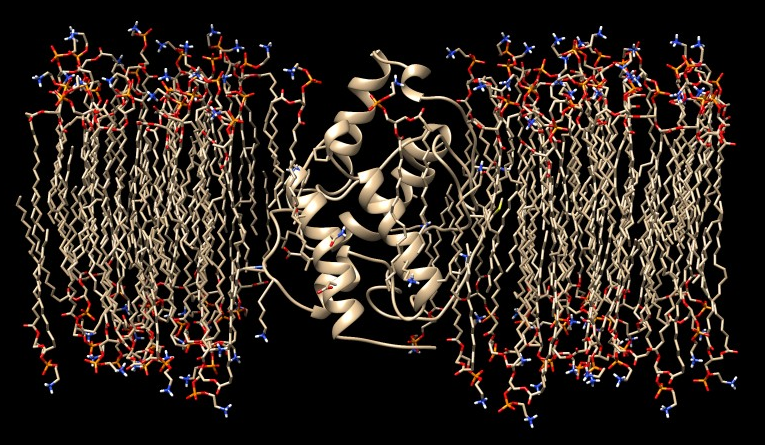


The relative large apparent hole in the membrane depends very much on the way one depicts the result. Only the backbone(s) (see above) or all atoms (see below).


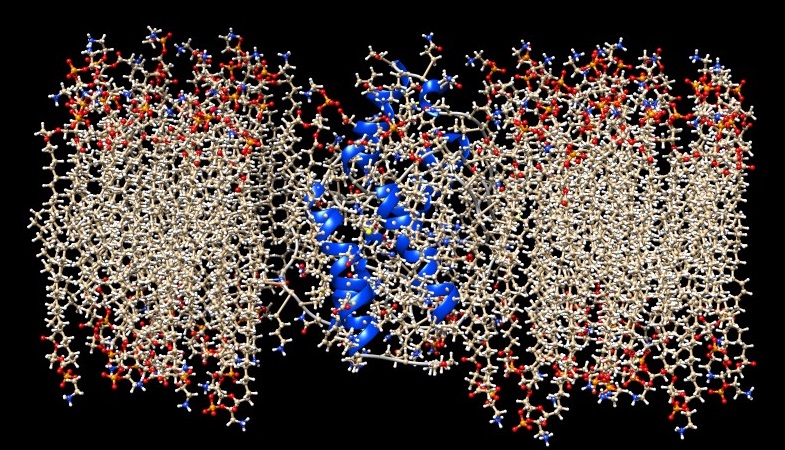


The MCPep results of the AA region of Pam18

The results of the wild-type AA 66-83 region and the region where the tyrosines (WT) were replaced by leucines are depicted in Fig. S1.:

Figure S1: MCPep results of AA 66-83 region.

WT-region:


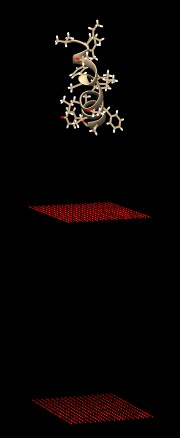


[
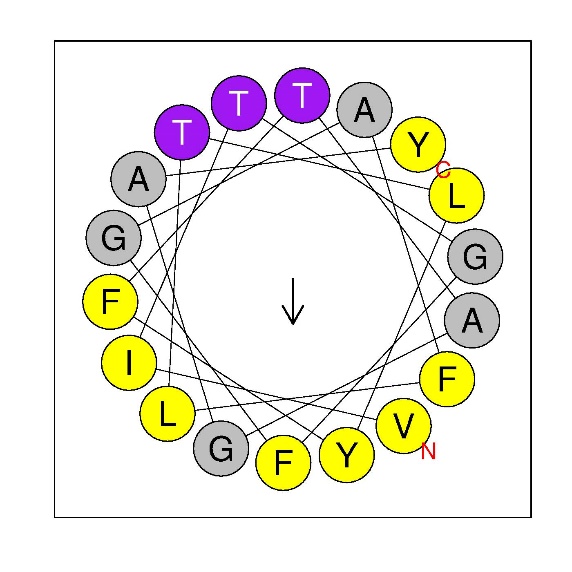
](http://heliquest.ipmc.cnrs.fr/tmpAmpH/AmpH-2932639292/PlotHelix-1.jpeg)

Region with two Leu:


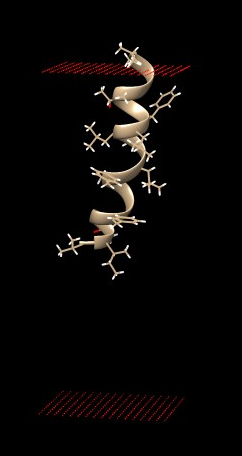


The TOPCON results of Pam18/Tim14

As described in the RESULTS section, the details belonging to the TOPCON analysis of Pam18 are depicted here:

Predicted topologies, predicted ΔG values and predicted distances to the membrane center (Z=0):


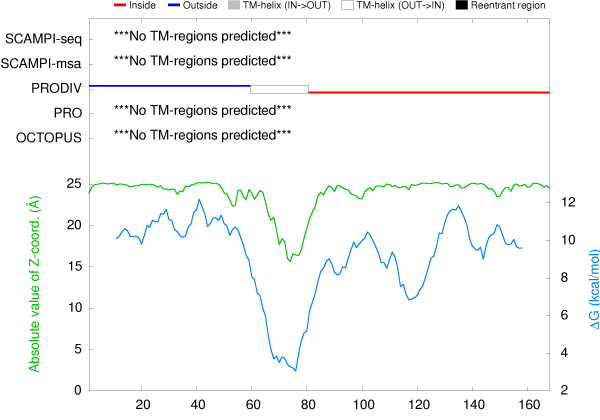


Predicted TM-helix positions:

|  |  |  |
| --- | --- | --- |
| SCAMPI-seq | | ***No TM-regions predicted*** |
|  | | |
| SCAMPI-msa | | ***No TM-regions predicted*** |
|  | | |
| PRODIV | | 1. 60-80 |
|  | | |
| PRO | | ***No TM-regions predicted*** |
|  | | |
| OCTOPUS | | ***No TM-regions predicted*** |
|  | | |
| TOPCONS | | ***No TM-regions predicted*** |
| Comparison of different hydrophobicity scales  The results of some of the most commonly used and best defined hydrophobicity scales are compared in Table S1. Some are known for their use in bioinformatics: Fauchere & Pliska (Heliquest), Eisenberg et al. (Hydrophobic Moment Plot), Kessel & Ben Tal (MCPep).  Table S1: Comparison of hydrophobicity scales (see for details indicated references).   \| Name \| ^a^Fauchere &  Pliska \| ^b^Eisenberg  et al. \| ^c^Kyte &  Doolittle \| ^d^Wimley &  White \| ^e^Kessel &  Ben-Tal \| \| --- \| --- \| --- \| --- \| --- \| --- \| \| Ala \| 0.310 \| 0.620 \| 1.800 \| 0.17 \| -0.2 \| \| Arg \| -1.010 \| -2.530 \| -4.500 \| 0.81 \| 19.8 \| \| Asn \| -0.600 \| -0.780 \| -3.500 \| 0.42 \| 7.7 \| \| Asp \| -0.770 \| -0.900 \| -3.500 \| 1.23 \| 11.5 \| \| Cys \| 1.540 \| 0.290 \| 2.500 \| -0.24 \| 0.4 \| \| Gln \| -0.220 \| -0.850 \| -3.500 \| 0.58 \| 5.4 \| \| Glu \| -0.640 \| -0.740 \| -3.500 \| 2.02 \| 9.5 \| \| Gly \| 0.000 \| 0.480 \| -0.400 \| 0.01 \| 0.0 \| \| His \| 0.130 \| -0.400 \| -3.200 \| 0.96 \| 6.8 \| \| Ile \| 1.800 \| 1.380 \| 4.500 \| -0.31 \| -2.6 \| \| Leu \| 1.700 \| 1.060 \| 3.800 \| -0.56 \| -2.6 \| \| Lys \| -0.990 \| -1.500 \| -3.900 \| 0.99 \| 7.4 \| \| Met \| 1.230 \| 0.640 \| 1.900 \| -0.23 \| 1.3 \| \| Phe \| 1.790 \| 1.190 \| 2.800 \| -1.13 \| -1.5 \| \| Pro \| 0.720 \| 0.120 \| -1.600 \| 0.45 \| 2.8 \| \| Ser \| -0.040 \| -0.180 \| -0.800 \| 0.13 \| 0.8 \| \| Thr \| 0.260 \| -0.050 \| -0.700 \| 0.14 \| 1.1 \| \| Trp \| 2.250 \| 0.810 \| -0.900 \| -1.85 \| 1.3 \| \| **Tyr** \| **0.960 (8th)** \| **0.260 (9th)** \| **-1.300 (10th)** \| **-0.94 (3th)** \| **4.3 (12th)** \| \| Val \| 1.220 \| 1.080 \| 4.200 \| 0.07 \| -1.2 \|   ^a^Fauchere, J., Pliska, V. (1983) Hydrophobic parameters p of amino-acid side chains from the partitioning of *N*-acetyl-amino-acid amides. *Eur. J. Med. Chem.* *8*, 369–375.  ^b^Eisenberg, D., Schwarz, E., Komaromy, M., Wall, R. (1984) Analysis of membrane and surface protein sequences with the hydrophobic moment plot. *J. Mol. Biol.* *15*, 125–142.  ^c^Kyte, J., Doolittle, R.F. (1982) A simple method for displaying the hydropathic character of a protein. *J. Mol. Biol.* *157*, 105-32.  ^d^Wimley, W.C., White, S.H. (1996) Experimentally determined hydrophobicity scale for proteins at membrane interfaces. *Nat. Struct. Biol.* *3*, 842-848.  ^e^Kessel, A., Ben-Tal, N. (2002). Free energy determinants of peptide association with lipid bilayers. In: Current Topics in Membranes. (Simon, S.A. & McIntosh, T. J., eds), vol. 52, pp. 205-253, Academic Press. | | |

The TOPCON results of Pam16

As described in the RESULTS section, the details belonging to the TOPCON analysis of Pam16 are depicted here:

Predicted topologies, predicted ΔG values and predicted distances to the membrane center (Z=0):


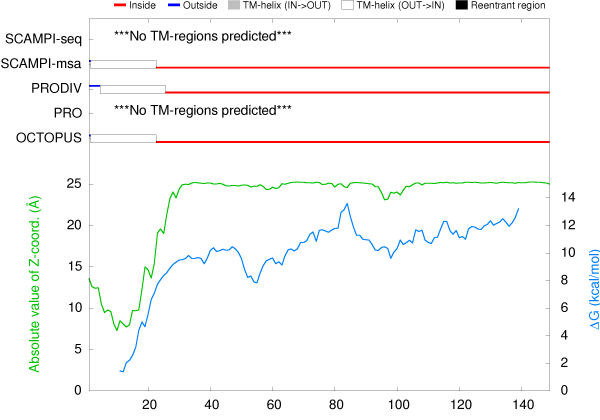


Consensus prediction (TOPCONS):

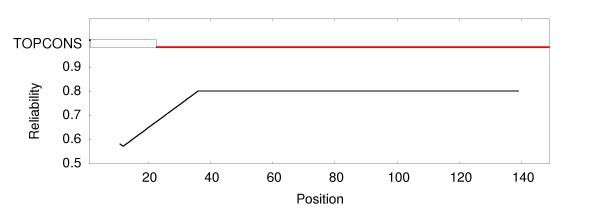


Predicted TM-helix positions:

|  |  |  |
| --- | --- | --- |
| SCAMPI-seq | | ***No TM-regions predicted*** |
|  | | |
| SCAMPI-msa | | 1. 2-22 |
|  | | |
| PRODIV | | 1. 5-25 |
|  | | |
| PRO | | ***No TM-regions predicted*** |
|  | | |
| OCTOPUS | | 1. 2-22 |
|  | | |
| TOPCONS | | 1. 2-22 |

Helical wheel plots of indicated lipid binding regions as obtained by Heliquest.

The residues in yellow correspond to hydrophobic amino acids, in purple polar residues are depicted. Blue and red coloured residues indicate charged residues (positive and negative respectively).

Figure S2:

Pam18(66-83):

[
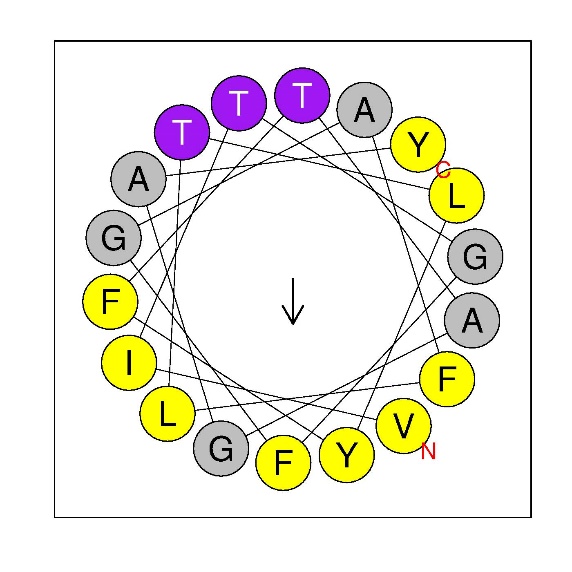
](http://heliquest.ipmc.cnrs.fr/tmpAmpH/AmpH-8753081643/PlotHelix-1.jpeg)

Pam16(5-22):

[
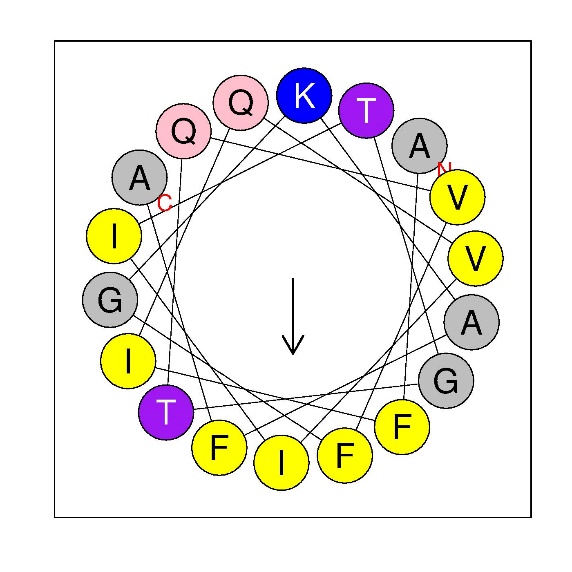
](http://heliquest.ipmc.cnrs.fr/tmpAmpH/AmpH-3263795970/PlotHelix-1.jpeg)

Pam17(52-69):

[
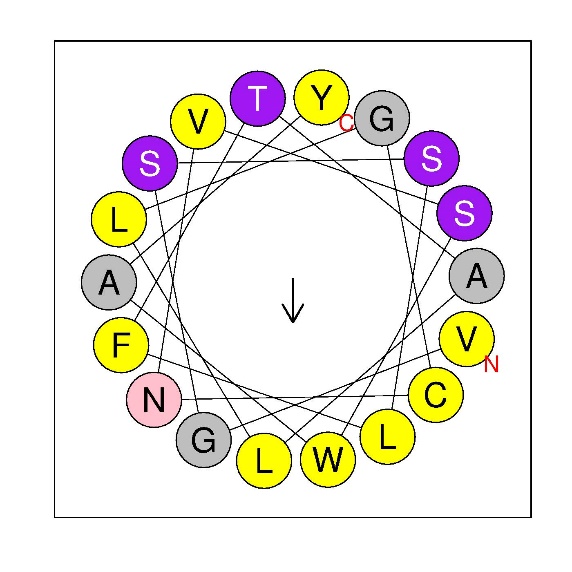
](http://heliquest.ipmc.cnrs.fr/tmpAmpH/AmpH-1720867540/PlotHelix-1.jpeg)

Pam17(87-104):

[
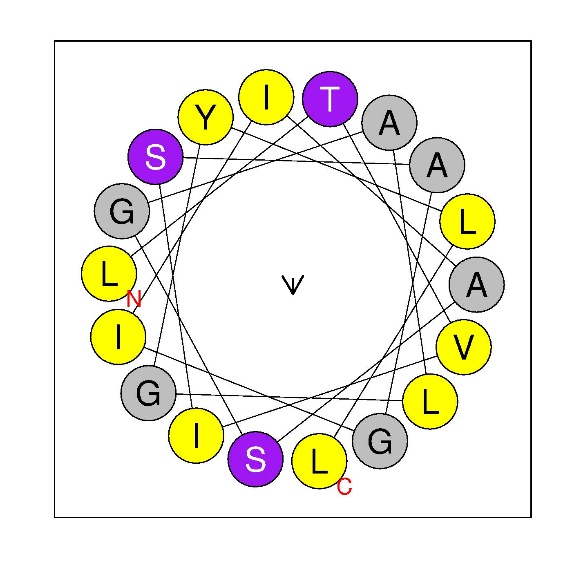
](http://heliquest.ipmc.cnrs.fr/tmpAmpH/AmpH-2507904922/PlotHelix-1.jpeg)

Tim44(301-318):

[
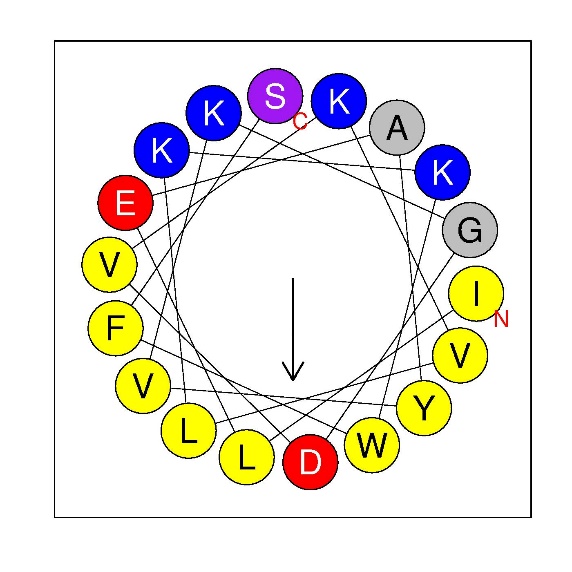
](http://heliquest.ipmc.cnrs.fr/tmpAmpH/AmpH-1481462041/PlotHelix-1.jpeg)
